# A SWOT analysis of translanguaging in healthcare education: overcoming language barriers for future professionals

**DOI:** 10.1186/s12909-025-08271-8

**Published:** 2025-12-12

**Authors:** Muhammad Alasmari, Obied Alaqlobi

**Affiliations:** Department of English Language & Literature, College of Arts and Letters, University of Bisha, Bisha, Saudi Arabia

**Keywords:** Healthcare education, SWOT analysis, Language barriers, Translanguaging

## Abstract

Translanguaging has emerged as a vital pedagogical strategy in multilingual healthcare education, where effective communication is essential. This study employs a SWOT analysis to assess the integration of translanguaging in preparing future healthcare professionals for linguistically diverse environments. Using a mixed-methods approach, it combines quantitative data from a survey of 90 medical students with qualitative insights from in-depth interviews with 10 students at various academic stages. Findings indicate that translanguaging enhances comprehension, promotes cultural competence, and fosters effective communication between patients and healthcare providers. Key opportunities include creating inclusive learning environments and improving healthcare accessibility for multilingual populations. However, challenges such as limited resources, inconsistent implementation, and educator resistance represent significant weaknesses. Threats include institutional reluctance and the potential over-reliance on native languages. Based on this analysis, the study offers strategic recommendations for educators and institutions aiming to implement multilingual practices in healthcare education, overcome language barriers, and better prepare future healthcare providers.

**Supplementary Information:**

The online version contains supplementary material available at 10.1186/s12909-025-08271-8.

## Introduction

In multilingual educational settings, translanguaging has emerged as a promising approach to support inclusive and effective learning. It can be defined as a dynamic process by which multilingual individuals draw on their full linguistic repertoire to make meaning, communicate, and learn [1–3]. Rather than switching between separate languages, it involves fluid language practices that reflect the speaker’s lived linguistic experience. It emerged as a response to the increasing need for understanding of how bilingual or multilingual individuals use their languages in fluid, dynamic ways. It has evolved into a pedagogical approach that leverages all linguistic resources available to students [4] [5]. contends that in higher education, where diverse students encounter language barriers that can hinder learning, translanguaging offers a means to enhance comprehension, engagement, and academic success. This is particularly relevant in fields like healthcare education, where effective communication across languages is crucial for both academic success and professional competence in real-world settings [6].

In healthcare education, the stakes are especially high, as language barriers can significantly impact the quality of healthcare that future professionals provide. During their training at the university, translanguaging allows them to access and express knowledge in multiple languages, thus fostering a deeper understanding of complex medical concepts. Despite its potential benefits, translanguaging in higher education is not without challenges [7]. pointed out some intricate issues of integration, such as the complexity of integrating multiple languages into standardized curricula, potential confusion among students, and resistance from educators accustomed to monolingual instruction, which require critical examination.

In Saudi Arabia, healthcare education is primarily conducted using English as the medium of instruction (EMI) [8–10], which is driven by the global applicability of English and the enhanced access to academic resources and career progression it offers [11]. English is recognized as the lingua franca in science and healthcare, and this enables Saudi medical students to gain international competence and engage with global developments [9]. However, there is keen interest in developing an Arabic version of the curriculum for future use, indicating consideration for bilingual approaches [12]. This suggests a growing awareness among decision-makers of the need to address language barriers in healthcare education [10] and to support students’ learning through the integration of Arabic.

### Research problem

Translanguaging, originally introduced as a pedagogical strategy that leverages students’ entire linguistic repertoire to enhance learning, has evolved into a broader decolonial philosophy that challenges traditional monolingual norms. In addition to fostering inclusivity [1, 3], it enhances clinical communication by allowing students to express complex ideas more authentically and accessibly. In healthcare, precise communication and the ability to convey complex medical concepts across different languages are crucial for ensuring patient understanding and safety.

Translanguaging in healthcare curricula holds promise for bridging linguistic gaps and fostering culturally competent care [13–15]. However, key questions remain about how effectively it prepares students for the multilingual and culturally diverse realities of the healthcare profession, where inclusive communication and patient-centered care are essential [6]. Empirical evidence on its actual impact remains limited. There is a critical need to evaluate its effectiveness in this domain. As clinical training environments are becoming linguistically diverse, there is growing recognition of the need for culturally responsive pedagogy. These trends highlight the importance of exploring how translanguaging can support multilingual learners and enhance communication outcomes. Addressing these gaps is necessary to determine whether translanguaging can improve education and practice, making it more responsive to diverse patient needs [13].

To better understand the practical implications of translanguaging in healthcare education, this paper homes in on translanguaging through a SWOT analysis of the insights gained from in-depth interviews with future healthcare professionals and survey data. It intends to identify the strengths, weaknesses, opportunities, and threats associated with translanguaging in healthcare programs in the context of Saudi Arabia. It dives into how translanguaging impacts students’ comprehension, communication skills, and cultural competence. It also identifies the institutional and pedagogical factors that support or hinder its implementation.

### Research questions

This study is an attempt to address the following research questions.

1. What are the perceived strengths of translanguaging in healthcare education?
2. What weaknesses or challenges do students and educators encounter when implementing translanguaging in medical training?
3. What opportunities does translanguaging offer for healthcare education?
4. What potential threats or risks could undermine the effectiveness of translanguaging in a healthcare setting?

## Literature review

### Theoretical foundations

Translanguaging is a critical area of inquiry within multilingual educational settings [4, 16, 17], especially in contexts where instruction is delivered in a language different from students’ first language (L1), such as in EMI and Content and Language Integrated Learning (CLIL) classrooms [9, 13]. The concept was first articulated by Cen Williams in the 1990 s to describe a pedagogical approach involving the strategic use of two languages for both input and output. It fosters bilingualism and biliteracy [1]. Over time, translanguaging has evolved into a broader theoretical framework that recognizes fluid and dynamic interplay of multiple languages, wherein bilinguals and multilinguals utilize their entire linguistic repertoire to effectively communicate and learn [1, 6, 18].

The theoretical foundations of translanguaging are grounded in the understanding of language as a flexible, integrated system rather than a set of discrete languages [1, 2, 5, 7]. The concept was expanded in 2014 to suggest that bilinguals do not merely switch between two separate language systems [7, 18]. Rather, they draw upon a unified linguistic repertoire that incorporates elements from all the languages that bilinguals know [1]. This perspective challenges conventional models of bilingual education, which often treat languages as isolated and classified. Instead, translanguaging is viewed as a means of leveraging all learners’ linguistic resources to enhance meaning-making and communication [6, 19].

Recent studies have also expanded the scope of translanguaging to include non-linguistic resources, such as gestures, visual aids, and other multimodal elements, recognizing their significance in the processes of communication and learning [2, 14, 20, 21]. This broader view, sometimes referred to as trans-semiotizing, shifts the focus from language per se to encompass a wide range of semiotic resources that individuals use for meaning-making [20]. The inclusion of these diverse resources underscores the complexity and richness of translanguaging as both a theoretical construct and a practical pedagogical tool. In healthcare classrooms, this may involve combining visual diagnostics with multilingual explanations to reinforce student comprehension.

### Translanguaging in education

Translanguaging is more than just a pedagogical approach. It serves as a critical framework that addresses power legacies embedded in language use and education. This perspective challenges traditional conceptions of discrete and bounded languages by recognizing the interconnected and fluid nature of linguistic practices [2]. introduced the notion of translanguaging as a communicative resource. It challenges colonial norms in education by encouraging teachers to move away from rigid, standardized language categories and instead embrace the diverse linguistic expressions and meanings their students bring. Historically, power structures have imposed rigid language boundaries, marginalizing indigenous and minority languages while privileging dominant languages.

Translanguaging provides a voice for multilingual students who are linguistically marginalized. It also acts as a counter-hegemonic approach that valorizes language epistemologies produced by Global South scholars [16]. By disrupting monoglossic ideologies and rejecting the separation of languages, translanguaging recognizes the interconnected linguistic repertoires of multilingual speakers. In contexts like Malaysia, for instance, translanguaging can challenge colonial language policies that reinforce rigid language boundaries and exclude local languages from educational discourse [22]. Similarly, in the Ryukyu Islands, incorporating translanguaging in healthcare training can help reclaim and revitalize local languages, resisting the assimilative pressures imposed by colonial and postcolonial state policies [4].

### Translanguaging: strengths and weaknesses

In pedagogical contexts, translanguaging offers numerous benefits, particularly in multilingual classrooms [5, 6]. It provides flexibility in teaching and learning, allowing students to engage with content in ways that are most meaningful to them [1, 23, 24]. It not only supports academic achievement but also affirms students’ linguistic identities, promoting a more inclusive and responsive educational environment. Empirical research has shown that translanguaging can enhance students’ comprehension of complex concepts by enabling them to draw on all their linguistic resources [25].

Despite its advantages, translanguaging poses some challenges. Implementing it in classrooms, particularly in EMI and CLIL settings [9, 25]. Where the primary goal is language acquisition and content learning, it can be a real challenge. Educators may struggle to balance the use of multiple languages while ensuring students develop proficiency in the target language. Institutional resistance to translanguaging can also emerge, particularly in educational systems that emphasize monolingual approaches [26] [14]. noted challenges such as educator hesitation and institutional norms favoring monolingual instruction.

### Translanguaging in higher education

Translanguaging plays a critical role in higher education [17, 21], particularly in contexts where students are trained for careers in healthcare [10]. In these settings, the ability to navigate multiple languages effectively is not only an academic skill but also a professional necessity. Healthcare professionals often work in multilingual environments where they must communicate with patients, colleagues, and other stakeholders in more than one language. Translanguaging, therefore, offers a pedagogical approach that allows students to use their entire linguistic repertoire to understand and engage with complex medical content [1]. It helps in bridging language barriers, enabling students to grasp intricate medical concepts in their native language and developing proficiency in the language of instruction (which is often English). As a result, students can comprehend the nuances of healthcare communication, which is essential for providing quality care in diverse linguistic settings [25].

In Saudi Arabia, where English is the primary language of instruction in medical and healthcare programs [3, 8, 9, 12, 18], translanguaging offers a way for navigating the tension between students’ native language, Arabic, and their professional development in English [12]. Studies such as [3, 8–10, 18] showed that Saudi medical students prefer code-switching between English and Arabic to improve their comprehension and communication skills. This reflects a localized form of translanguaging that aligns with their linguistic needs. However, adopting English as the medium of instruction has led to resistance from educators and institutions who fear that reliance on Arabic could hinder students’ proficiency in medical English, which presents a unique contextual challenge [27].

When it comes to inclusivity, translanguaging in healthcare-related programs in Saudi Arabia [3, 10] supports the academic success of multilingual students. By allowing students to use all their linguistic resources, translanguaging creates a more accessible and equitable learning environment. This is especially important in healthcare education, where the stakes are high [10], and the ability to communicate effectively can have life-and-death consequences [10, 26] [1]. showed that when students are allowed to switch between languages, they are more engaged, perform better academically, and are more confident in their abilities to apply their knowledge in real-world settings.

As such, translanguaging represents a paradigm shift in our understanding of language use in education [2, 15, 17, 21]. It offers a flexible and inclusive approach to teaching and learning that recognizes the full linguistic repertoire of students. Although it fosters better communication in multilingual healthcare environments and enhances the cultural competence of future healthcare providers [2], it requires thoughtful consideration of the challenges it poses. Continued research in higher education contexts is essential for refining the concept and exploring its practical applications in multilingual educational settings, such as healthcare education [10, 27]. The current study set out to enrich research on translanguaging with data collected in the Saudi context. It examines how translanguaging plays a transformative role by enabling students and professionals to draw on their entire linguistic and semiotic repertoire in healthcare education. The study is based on the premise that language is fluid and interconnected. With this view in mind, healthcare educators can create more inclusive learning settings, thereby improving both the educational experience of students and the quality of healthcare provided to the patients.

## Methodology

This study employed a mixed-methods approach to address the research questions. It applies a SWOT analysis to examine the pedagogical role of translanguaging in multilingual healthcare education. It utilizes quantitative surveys and qualitative interviews with medical students to identify and evaluate the strengths, weaknesses, opportunities, and threats of translanguaging in the context of Saudi Arabia. This research approach allows for an in-depth understanding of key themes and patterns. Employing a SWOT framework ensured a comprehensive analysis of the internal and external factors that influence translanguaging.

### Data collection

#### Questionnaire

Data was gathered through a two-step process comprising a survey and follow-up interviews. The survey was distributed to 120 medical students, with 90 completed responses received, yielding a response rate of 75%. It aimed to obtain quantitative insights into the role of Arabic alongside English in healthcare education. It included Likert-scale items assessing students’ perceptions of comprehension, learning support, communication challenges, and engagement. Table 1 outlines the thematic description of survey questions, and a copy of the final questionnaire is included as supplementary material (Appendix A).

**Table 1:** Thematic description of survey questions

Item Code	Descriptive Name	Survey items
Q1	Comprehension Enhancement	“Using Arabic alongside English in healthcare education enhances students’ comprehension of complex medical concepts and specific terminology.”
Q2	Learning Support Without Barriers	“Using Arabic for medical management and instructions in healthcare education provides an opportunity to support students’ learning without language barriers.”
Q3	Oral Communication Limitation	“Relying on Arabic for answering oral questions during healthcare education may limit students’ ability to develop medical communication skills in English.”
Q4	Written Proficiency Hindrance	“Allowing students to use Arabic for written communication could hinder their proficiency in writing medical terms in English.”
Q7	Motivation to Learn	“Incorporating Arabic in using medical terms enhances students’ motivation to learn and engage more deeply with the material.”
Q8	Understanding Course Content	“Integrating Arabic in medical training improves students’ overall understanding of the subject matter and course content.”
Q9	Mastery of Medical Terminologies in English	“Excessive use of Arabic in medical interns may limit students’ ability to master medical terminologies in English, potentially affecting their professional communication skills.”
Q10	Engagement with English	“Students’ comfort in using Arabic may reduce their willingness to engage with English, which could be a disadvantage in their future medical careers where English is dominant.”

Before participation, informed consent was obtained from all students. The questionnaire was provided in English, with oral translation offered to participants who needed clarification to ensure clarity and consistency. The translation was standardized by using a pre-approved script and trained research assistants who followed a uniform protocol for clarifying survey items in Arabic. A pilot version of the survey was tested on a small group (*n* = 10) with similar characteristics to the target population to evaluate clarity, relevance, and structure. Based on the pilot results, items Q5 and Q6 were removed (from Table 1) due to a lack of alignment with the study objectives.

#### Interviews

Following the survey, in-depth interviews were conducted with future healthcare professionals to gain a better understanding of translanguaging in higher education, viz., within the context of healthcare education. The participants were 10 male medical students in different academic years, attending a midsize university in Saudi Arabia with a focus on medical practice. This diversity in academic stages provided more understanding of how translanguaging is experienced and perceived at various stages of medical training. Their perspectives offer valuable insights into the practical applications of translanguaging in enhancing communication, cultural competence, and readiness for real-world medical environments.

The interview questions were designed to explore participants’ experiences, perceptions, and challenges related to translanguaging in their training and anticipated professional environments. The interview questions were developed in alignment with the survey items and insights from prior studies examining opportunities and challenges in translanguaging [3, 8, 10, 14]. They were refined through expert validation to ensure clarity and alignment with the research questions and objectives. Two faculty members with expertise in language education and healthcare pedagogy served as expert validators. They assessed the interview questions based on these criteria: clarity, relevance to the research questions, cultural appropriateness, and alignment with the study’s objectives.

**Fig. 1:**
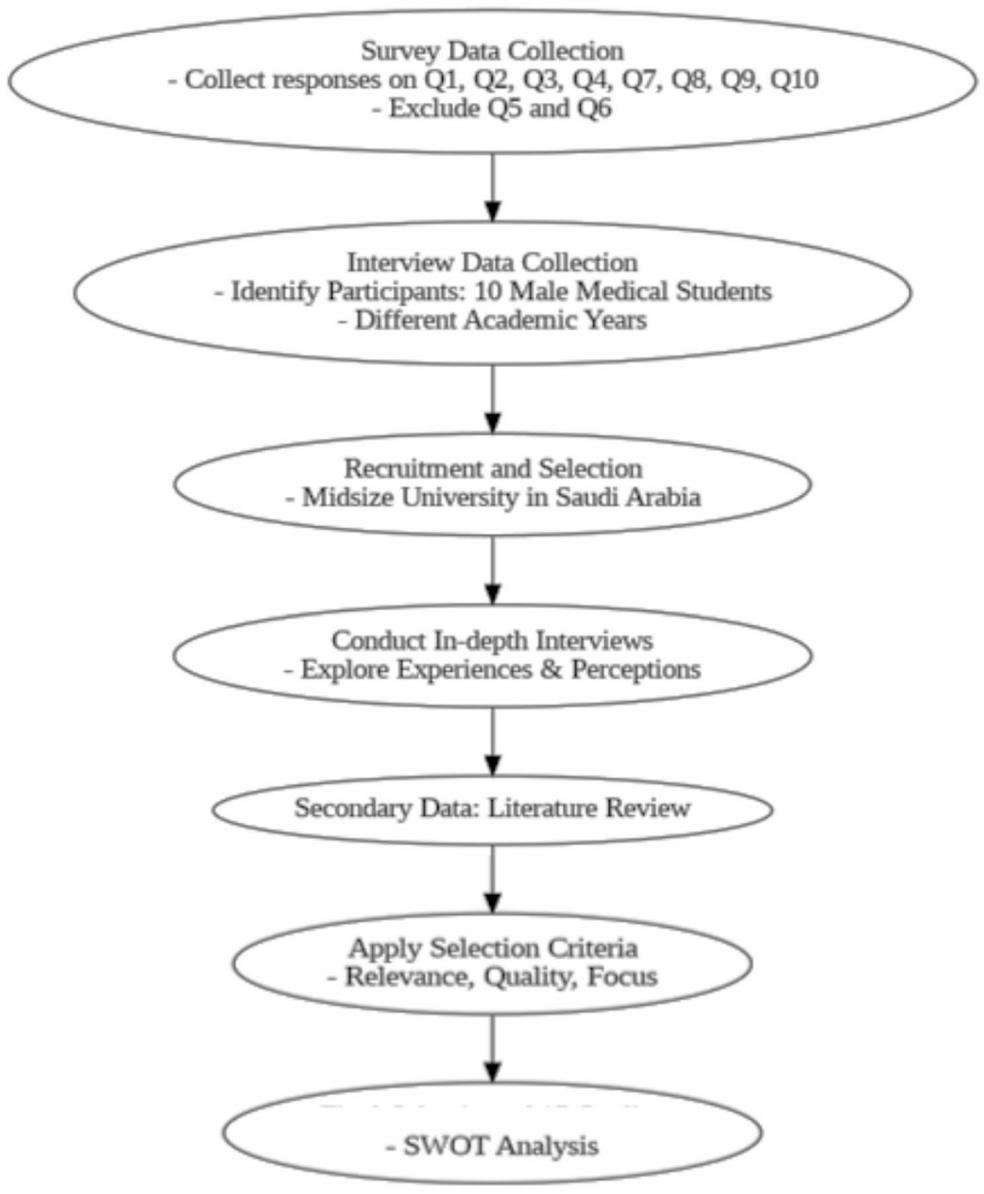
Overview of data collection and analysis

The overall process of data collection is displayed in Fig. 1, in which the SWOT framework synthesizes insights from all data sources. SWOT stands for strengths, weaknesses, opportunities, and threats associated with translanguaging in healthcare education.

### Data analysis

Data analysis for this study involved a systematic examination of the collected interview data and selected literature using a qualitative approach guided by the SWOT framework. The interview dataset was transcribed and thematically analyzed, using [23]’s framework for thematic analysis. The process began with familiarizing the dataset, in which the transcripts were read multiple times to understand the content. Initial codes were then generated to capture key features of the data and were subsequently organized into potential themes. These themes were reviewed for internal consistency and relevance to the research questions, then clearly defined and named to reflect their core meanings.

The SWOT framework, acknowledged in several prior studies (e.g [28]). is supportive of strategic planning and decision-making by offering a structured approach to evaluating stakeholders’ perspectives. It provided a holistic framework for understanding how these practices are perceived and applied across various stages of medical training in the context of this study. It facilitated a balanced examination of translanguaging in healthcare education, addressing both internal factors (strengths and weaknesses) and external factors (opportunities and threats) that influence its effectiveness. The analysis focuses on overcoming language barriers and enhancing the professional readiness of future healthcare providers.

## Results and discussion

The survey findings and interview themes are presented in alignment with the research questions. They are organized into key themes related to translanguaging in the healthcare curriculum within the Saudi context. The strengths, weaknesses, opportunities, and threats of translanguaging are discussed in relation to existing literature, which serves to contextualize and reinforce the findings derived from this investigation. Results pertinent to each of the research questions are outlined first. Then they are discussed in relation to previous studies and literature.

RQ1 What are the perceived strengths of translanguaging in healthcare education?

To answer this question, the interview data with medical students, supported by the review of selected literature, highlight several key strengths of translanguaging in the context of this study. The key themes relevant to the strengths include communication enhancement and cultural competence.

### Enhancing communication and Understanding

Translanguaging can improve communication between healthcare providers and patients in multilingual environments, which contributes to improved healthcare service. Effective communication ensures that patients fully comprehend their diagnosis, treatment options, and follow-up instructions. By integrating multiple languages and dialects into interactions, translanguaging allows healthcare professionals to engage patients in a way that bridges language barriers and enhances understanding. For instance, one participant reported that translanguaging simplifies complex concepts, allowing them to understand and articulate ideas more accurately. Another participant mentioned that it conveys the idea correctly. A third one added “we can understand the sentence logically and in the correct meaning”. This is particularly valuable when patients are not proficient in the dominant language used by the healthcare system.

In healthcare systems where a significant portion of the population speaks languages other than the dominant language, such as Spanish in the United States, translanguaging offers healthcare providers the flexibility to switch between languages and dialects to communicate effectively with patients. It ensures that critical medical information is conveyed accurately, reducing the risk of miscommunication and medical errors. Furthermore, it allows healthcare providers to use patient-centered language, making complex medical terms more accessible and easier to understand. This fosters trust and cooperation between healthcare providers and patients, as observed in classroom settings where the participants reported that they found it easier to engage when teachers alternated between languages: “When the teacher switches between English and Arabic, everyone can contribute and feel included in the discussion” Saudi medical students showed a strong preference for code-switching between English and Arabic for course comprehension, enhancing understanding of complex concepts.

### Fostering cultural competence

**Fig. 2:**
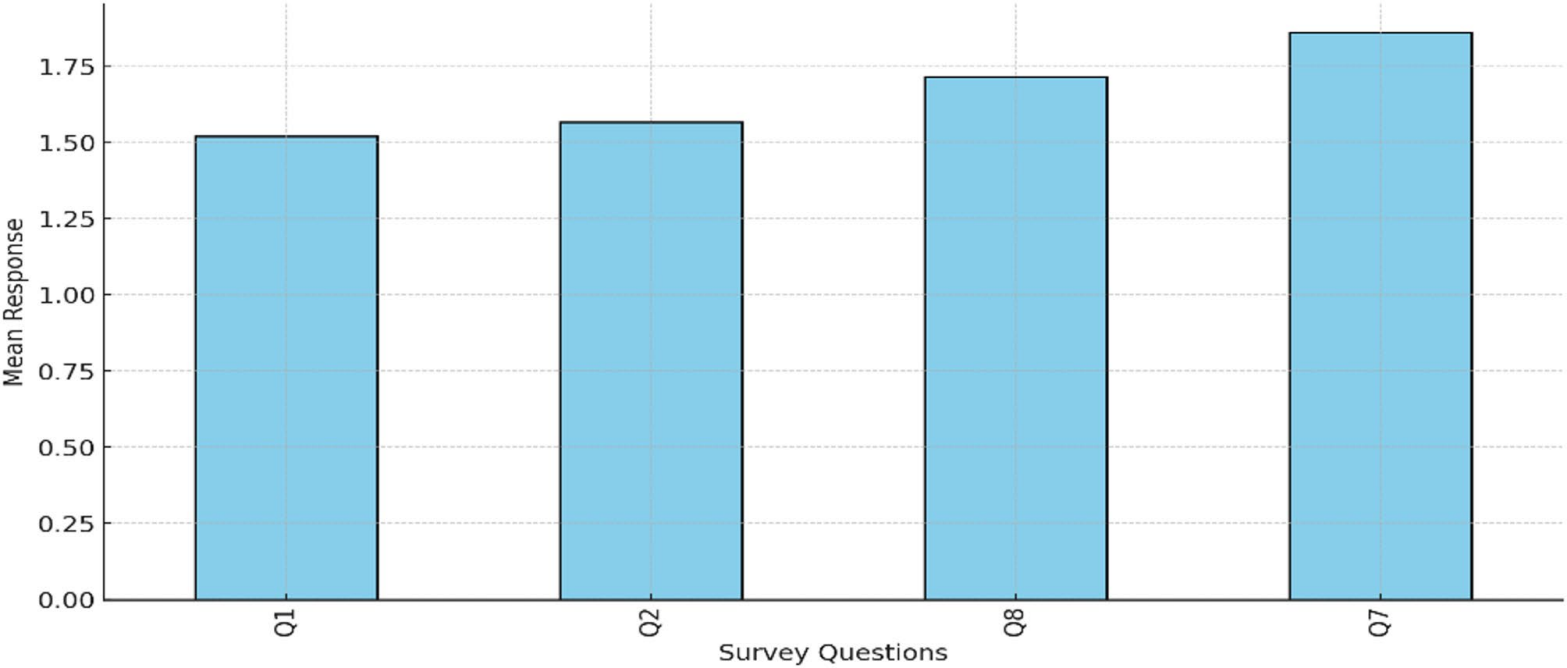
Students’ responses to aspects of enhancement, support, motivation, and course content

Strengths of translanguaging, as stemmed from the survey, include participants’ perceptions of translanguaging. They stated that it enhances their comprehension of complex medical concepts and terminology, with a mean score of 1.52 for the survey item related to comprehension enhancement. Similarly, the item on learning support without barriers received a mean score of 1.57, indicating that students valued the use of Arabic alongside English in creating a supportive learning environment. Figure 2 illustrates the overall spread and central tendencies of the data, providing a clearer understanding of how students perceived these strengths.

Moreover, students reported a positive impact on understanding course content and motivation to learn, with mean scores of 1.72 and 1.86, respectively. A One-Way ANOVA confirmed statistically significant differences between these items, indicating that students consistently perceived translanguaging as beneficial across different dimensions of their education (p-value = 0.039). This indicates that students rated certain aspects, such as comprehension and motivation, more positively than others, confirming that translanguaging has varied but meaningful impacts on different dimensions of healthcare education.

**Fig. 3:**
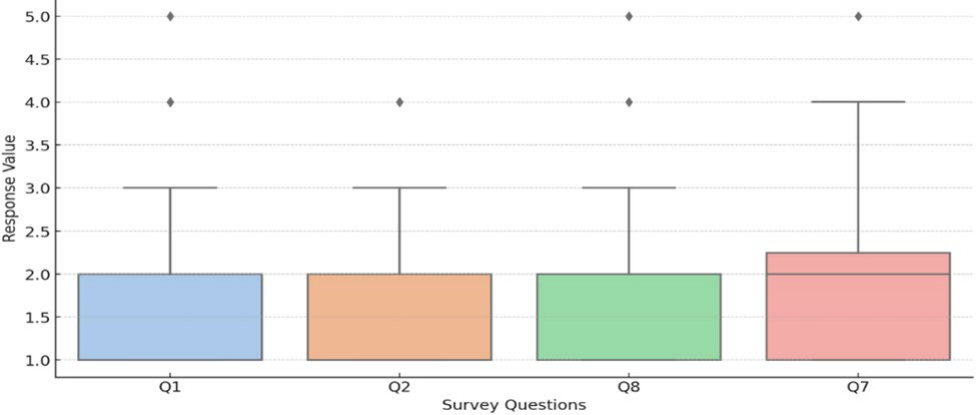
Student responses to the benefits of translanguaging

Figure 3 highlights the mean responses for each of these items, showcasing their relative significance in the overall perception of strengths. In healthcare, cultural competence is essential for addressing the unique health beliefs and practices of different cultural groups. For example, some patients may have specific religious or cultural beliefs that influence their approach to illness and treatment. Translanguaging allows healthcare providers to recognize and accommodate patients’ cultural beliefs by using language that aligns with the patient’s cultural context, fostering a more inclusive and supportive healthcare environment.

These findings align with insights from the existing literature that highlight the value of translanguaging in enhancing communication, engagement, and understanding [13]. maintain that translanguaging fosters cultural competence among healthcare professionals. Cultural competence in this study refers to the ability of healthcare providers to deliver care that is respectful of and responsive to the cultural and linguistic needs of patients. Translanguaging facilitates this by allowing healthcare providers to incorporate cultural nuances into their communication, ensuring that patients from diverse backgrounds feel understood and respected [14]. In a classroom context, observations where translanguaging helped students express themselves more confidently and understand lessons better were evident in [13] in which teachers switched languages to accommodate students’ linguistic needs. It helped them clarify their thoughts and feel more secure in expressing themselves. Additionally, translanguaging enables healthcare providers to engage with patients on a deeper level by acknowledging the broader cultural significance of language, thus improving patient-provider relationships and enhancing patient satisfaction [29].

RQ2 What weaknesses or challenges do students and educators encounter when implementing translanguaging in medical training?

The second research question relates to the weaknesses of translanguaging in healthcare education. A key weakness that emerged from this analysis is the difficulty of implementing it effectively across healthcare systems. Healthcare institutions often lack the necessary resources, such as trained multilingual staff, translators, and educational materials, to fully integrate translanguaging into everyday practice. This gap can hinder the widespread adoption of translanguaging, particularly in regions where healthcare systems are already stretched thin. Furthermore, students’ responses indicate that using Arabic makes it easier for them to convey and accept information, but it may have a negative effect on improving English skills, as we may rely too much on Arabic. This demonstrates how a similar dynamic might arise in healthcare contexts, where reliance on the dominant language could hinder the development of necessary multilingual skills.

Moreover, translanguaging requires healthcare providers to undergo additional training to become proficient in using multiple languages and understanding the cultural nuances associated with those languages. This can place additional burdens on healthcare professionals, who are often already overwhelmed by their clinical responsibilities.

### Inconsistent application across medical settings

Another weakness of translanguaging that surfaced from the analysis is the risk of inconsistent application across different healthcare institutions. While some healthcare providers may be well-versed in translanguaging, others may lack the necessary training or institutional support to effectively implement these strategies. Translanguaging addresses inconsistencies in care by allowing healthcare providers to communicate with patients in their preferred language or dialect, ensuring that vital medical information is conveyed accurately. In educational settings, this inconsistency was reflected in student frustrations when translanguaging was not employed effectively. For example, one student expressed feeling confused when the teacher did not use translanguaging: “I feel confused in class when the teacher doesn’t use any Arabic.” In a healthcare context, similar inconsistencies could result in communication breakdowns that negatively impact patient care [27].

**Fig. 4:**
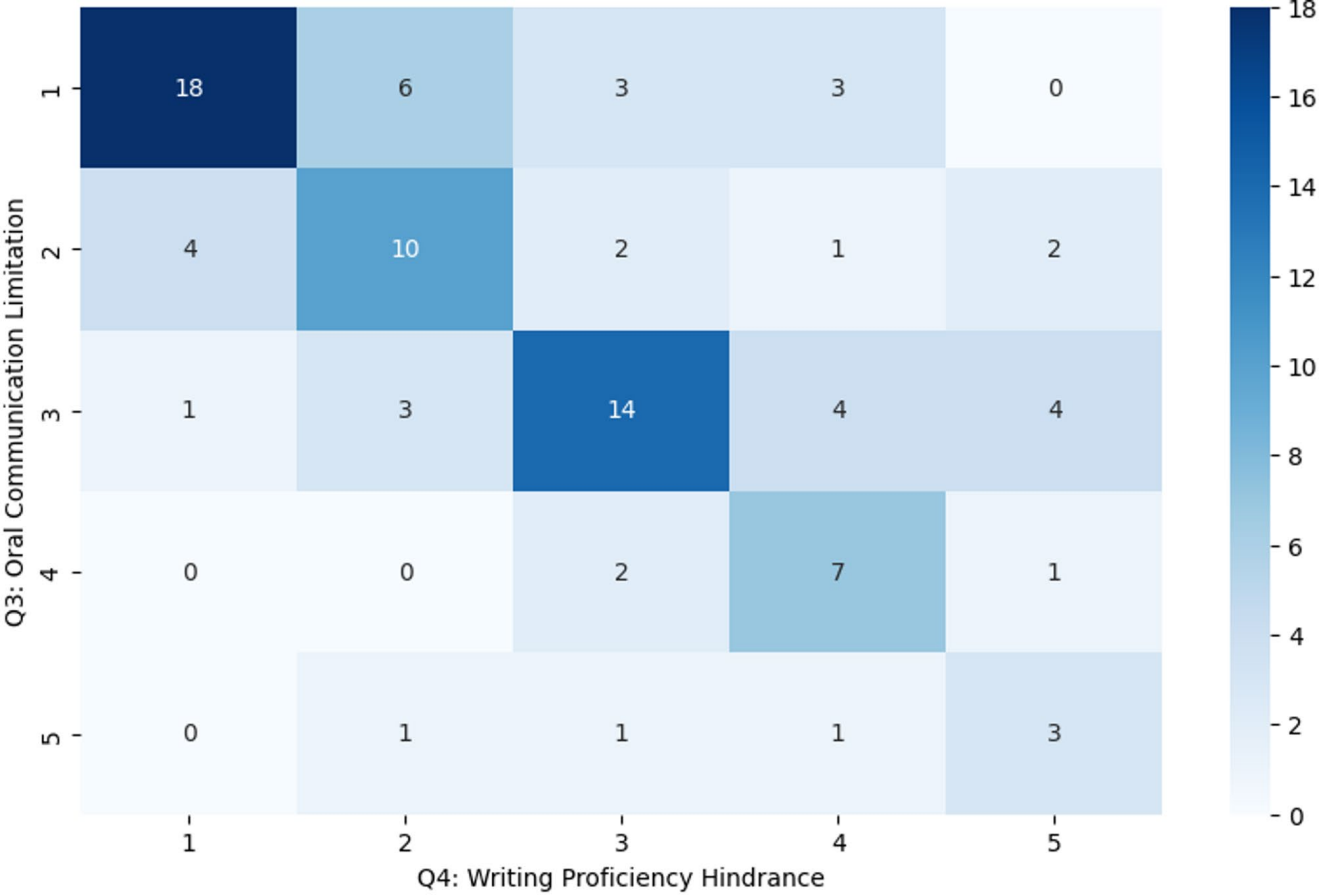
Association between students’ perceptions of oral communication limitations and difficulties in writing proficiency

Evidence of weaknesses also includes those elicited from the survey, providing additional quantitative insights into the weaknesses of translanguaging in healthcare education. Q3 focused on perceived limitations in oral communication skills when relying on Arabic, and Q4 addressed the hindrance to proficiency in writing. A cross-tabulation of responses to these two items revealed patterns of agreement, suggesting that students who identified limitations in one area often recognized challenges in the other. The results of a Chi-Square Test of Independence indicated a statistically significant association between Q3 and Q4 (χ² = 78.99, *p* = 2.53e-10), showing that students who perceived limitations in oral communication also tended to report hindrances in writing proficiency. Figure 4 illustrates this association, with darker colors indicating a higher frequency of combined responses. This supports the notion that translanguaging can present broader challenges to the development of essential English communication skills, potentially impacting students’ ability to function effectively in multilingual healthcare environments.

In the literature, one of the challenges identified is the language barrier faced by students in understanding and retaining complex medical terminologies in English, especially when it is not their first language [27]. This difficulty affects their ability to grasp key healthcare concepts and effectively communicate in professional settings. In addition, translanguaging may not always align with institutional priorities or policies, particularly in healthcare systems that emphasize efficiency over personalized care. In some settings, the pressure to see more patients in less time may discourage healthcare providers from engaging in the more time-consuming practice of translanguaging, resulting in its underuse or misuse [14]. This can reduce the effectiveness of translanguaging as a tool for improving communication and cultural competence in healthcare. Besdies [15], asserted that without adequate support, such as professional development opportunities and access to linguistic resources, the full potential of translanguaging may not be realized.

RQ3 What opportunities does translanguaging offer for healthcare education?

Results revealed some opportunities that translanguaging offers to health care programs. On top of that are the accessibility of healthcare and inclusive healthcare environments.

### Increasing accessibility of healthcare

The increased accessibility of healthcare services for patients who do not speak the dominant language is advantageous. Language barriers can significantly impede patients’ ability to access healthcare, understand medical information, and adhere to treatment plans. Translanguaging addresses this issue by allowing healthcare providers to communicate with patients in their preferred language or dialect, ensuring that vital medical information is conveyed accurately.

Furthermore, in educational settings, translanguaging has been shown to accelerate the pace of learning for students with low English proficiency, enhancing their understanding of key concepts, as one student reported that “This method helps in understanding and speeds up the comprehension of the material”. This demonstrates how translanguaging can similarly be applied in healthcare settings to make vital information more accessible to patients.

This approach can help reduce disparities in healthcare access for minority language speakers and marginalized groups. When healthcare providers switch between languages and use culturally familiar terms, patients feel more comfortable and understood, which in turn increases their willingness to seek care and follow medical advice. This opportunity to make healthcare more accessible is particularly important in diverse populations, where linguistic diversity is often a barrier to receiving timely and appropriate care.

For example, it allows students to discuss clinical scenarios in both their home language and the language of instruction, which enables deeper reflection and more nuanced understanding. In one case, a student used Arabic to explain a culturally specific health belief, which enriched the group’s discussion and improved their ability to communicate with patients from similar backgrounds. These opportunities can improve communication, enhance cultural understanding, and increase the accessibility and quality of care for multilingual populations. The key opportunities of translanguaging for healthcare education are outlined below.

### Promoting inclusive healthcare environments

Survey data included supportive evidence on two key opportunities associated with translanguaging: learning support without barriers and motivation to engage more deeply with the material. These were assessed using survey items Q2 and Q7, which measured students’ perceptions of learning support and motivation, respectively. Descriptive statistics showed that students had a slightly higher mean agreement with statements related to motivation (mean = 1.84) compared to learning support (mean = 1.58). The variability in responses, as indicated by the standard deviations, suggested that participants held somewhat diverse views on these opportunities.

**Fig. 5:**
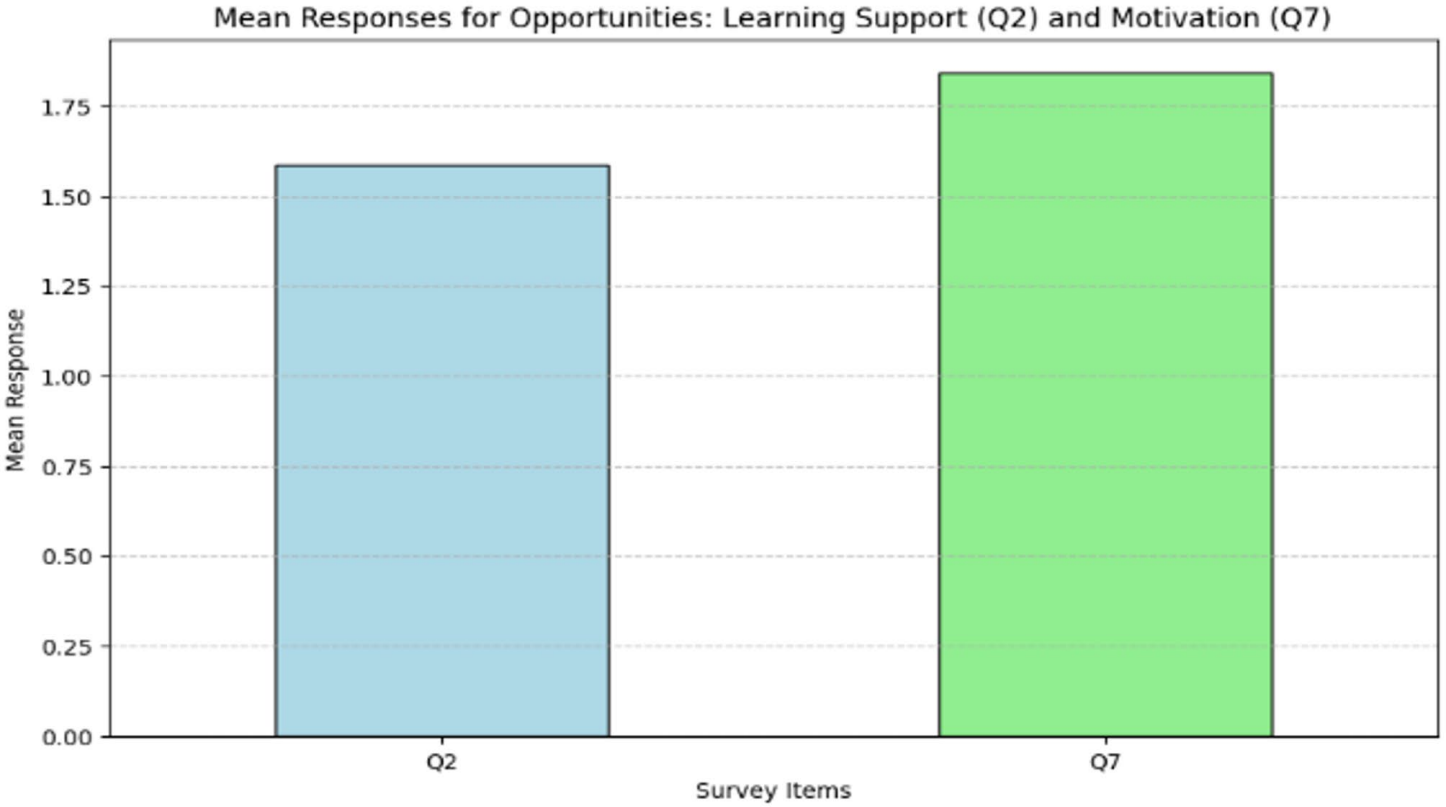
Mean responses for perceived opportunities: learning support (Q2) and motivation (Q7)

To further explore these perceptions, a One-Way ANOVA was conducted to test if there was a significant difference between the mean responses for learning support and motivation. Figure 5 illustrates the average levels of agreement for these two items, highlighting their relative importance in the context of translanguaging in healthcare education. Students perceived the benefits of translanguaging for learning support and motivation to be relatively similar. The notion that translanguaging provides a balanced opportunity to enhance both learning support and motivation reinforces its role in creating more accessible and engaging educational environments. Although the difference yields a p-value of 0.065, it does not meet the conventional threshold for statistical significance, due to the small sample, and it may indicate a non-significant trend.

In this regard, previous findings show interest in developing an Arabic curriculum for healthcare education [12], which presents an opportunity to create more accessible and culturally inclusive learning environments. In line with that [13], contends that translanguaging also presents an opportunity to create more inclusive healthcare environments that cater to the needs of diverse patient populations. By integrating multiple languages into everyday medical interactions, healthcare providers can foster a sense of inclusion and respect for linguistic and cultural diversity. This approach not only enhances patient experience but also helps build stronger relationships between healthcare providers and patients, leading to better health outcomes [14]. Also, translanguaging creates a space where all participants, regardless of their language proficiency, can contribute and feel included, as one student reported that “When the teacher switches between English and Arabic, everyone can contribute and feel included in the discussion”. This inclusivity is essential for creating healthcare environments where patients feel comfortable expressing their concerns, thus improving patient trust and engagement.

 [29] contends that inclusive healthcare environments are essential for promoting patient trust and engagement, particularly among minority language speakers who may feel marginalized in traditional healthcare settings. Translanguaging allows healthcare providers to communicate in a way that respects the patient’s cultural identity, making patients feel more valued and respected. This, in turn, can improve patient satisfaction, increase adherence to treatment plans, and ultimately lead to better health outcomes.

RQ4 What potential threats or risks could undermine the effectiveness of translanguaging in a healthcare setting?

This question probes into threats to translanguaging that could undermine its effectiveness. These potential challenges may affect the successful integration of translanguaging into healthcare systems. The fourth research question focused on identifying threats, addressed through an analysis of survey data. It highlighted potential threats associated with translanguaging, including the risk of over-reliance on the first language, which may limit students’ ability to effectively use medical terminologies in English. This was assessed through two specific survey items: Q9, which focused on students’ perceptions of limitations in mastering medical terminologies, and Q10, which addressed their reduced willingness to engage with English.

**Fig. 6:**
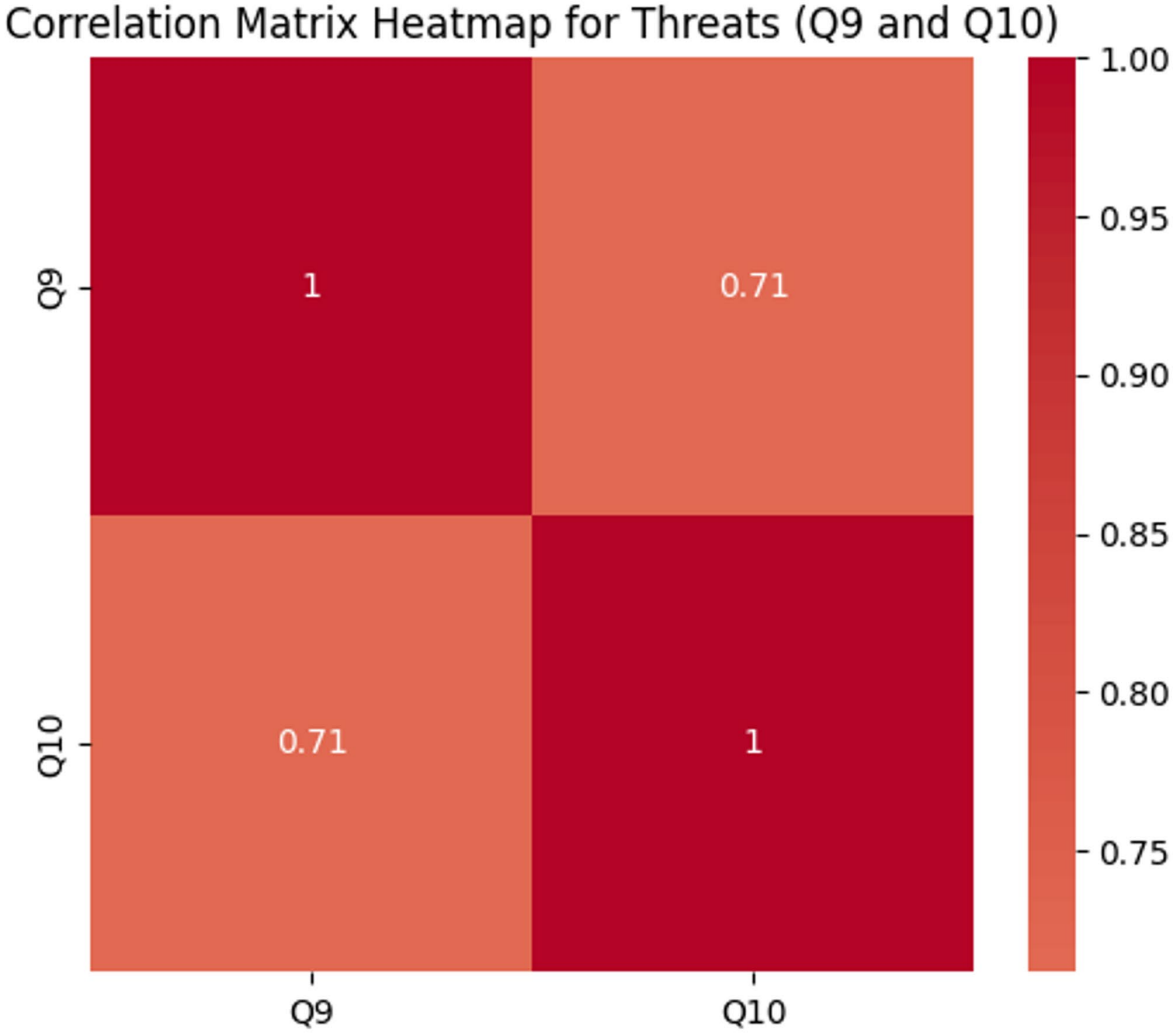
Relationship between perceived communication barriers and cultural misalignment

A correlation analysis revealed a strong positive relationship (Spearman correlation = 0.71, *p* < 0.001) between these two items. This indicates that students who reported greater limitations in mastering English medical terminologies due to translanguaging also expressed a reduced willingness to engage with English. The strong positive correlation suggests that students who experience difficulties in mastering medical terminology in English are more likely to feel disengaged from using English. This could have significant implications for healthcare education, as insufficient development of English communication skills may hinder students’ ability to effectively communicate with patients and colleagues in real-world healthcare environments. The results emphasize the need for balanced translanguaging that encourages students to strengthen both their native language and English proficiency. Figure 6 illustrates the correlation between these two items, showing a strong positive relationship:

This quantitative data further expands the understanding of translanguaging’s potential risks by providing evidence of a significant correlation between students’ perceived limitations in mastering medical terminologies and their reduced willingness to engage with English. These findings highlight the importance of carefully balancing native language use with the development of essential English skills in healthcare education. While the statistical associations between survey items, such as the strong correlation between limitations in mastering English medical terminology and reduced willingness to engage with English, are informative, their practical significance lies in how they can guide curriculum design and pedagogical strategies. These relationships suggest that students who rely heavily on their first language (Arabic) may struggle to develop proficiency in English medical discourse, which is essential for global healthcare communication.

### Resistance from educators and institutions

In the existing literature on translanguaging, as in the context of the study at hand, potential resistance from educators, administrators, and institutions is a potential threat. Many healthcare professionals have been trained in traditional, monolingual approaches to communication, and the shift to a translanguaging model may be met with skepticism or reluctance. For instance, one student voiced concerns about relying too much on their first language (L1) in English classes, fearing it might hinder the development of second language (L2) proficiency: “Using Arabic in English classes may prevent students from practicing speaking in English.“​.

### Inconsistent application and quality of care

The participants expressed frustration when translanguaging was inconsistently applied. One student reported feeling unsure about the lesson when the teacher did not use their first language: “I feel confused in the class when the teacher doesn’t use any Arabic.“​ In healthcare, this inconsistency could lead to communication breakdowns, adversely impacting patient care. While some healthcare providers may fully embrace translanguaging, others may lack the training, resources, or institutional support to use it effectively. This can result in a fragmented healthcare system where the quality of communication and care varies significantly depending on the location or provider.

Furthermore, translanguaging may be underutilized in settings where there is pressure to prioritize efficiency over patient-centered communication. In such environments, healthcare providers may not have the time to engage in the nuanced, language-rich interactions that translanguaging requires, leading to rushed or incomplete communication. This inconsistency could undermine the goals of translanguaging, which aim to improve patient-provider relationships and enhance care for diverse patient populations. Some students worry that over-reliance on L1 during training might negatively affect their ability to use medical terminologies in real-world healthcare settings: “If I learn these terms in Arabic, how can I use them in a hospital setting with colleagues who don’t speak Arabic?“​.

Putting the findings of the four research questions in the context of literature, some educators may view translanguaging as overly complex or impractical, particularly in fast-paced medical environments where clear and concise communication is often prioritized [15, 24]. This concern is reflected in educational settings, where students and educators have expressed reservations about the use of translanguaging in certain contexts. Resistance might arise in healthcare environments where communication in L2 is essential. In addition, institutions may resist implementing translanguaging due to the perceived costs and logistical challenges. Developing curricula that support translanguaging requires significant investment in training, resources, and support systems. Without institutional buy-in and the necessary funding, translanguaging may remain underutilized, limiting its potential impact on healthcare education [14].

The integration of multiple languages in healthcare settings allowed students to grasp complex medical concepts more effectively, facilitating clearer patient-provider interactions and improving overall healthcare outcomes [24]. Additionally, interview data showed that translanguaging helped bridge cultural gaps and promoted sensitivity, enabling healthcare providers to connect more deeply with patients from diverse backgrounds [29]. These findings were consistent with the literature review, which highlighted translanguaging’s ability to enrich the educational experience and foster more inclusive communication. Similarly, the study of [3] provides similar insights into translanguaging as it enables learners to access academic content more effectively by leveraging their full linguistic repertoire.

Taken as a whole, the survey and interview provide valuable insights into the strengths, weaknesses, opportunities, and threats associated with translanguaging in healthcare education, demonstrating the capacity of translanguaging to enhance communication and foster cultural competence. The study revealed potential challenges and threats. The quantitative analysis of survey responses indicated a strong positive correlation between students’ perceptions of limitations in mastering medical terminology and their reduced willingness to engage with English. This correlation highlights the risk of over-reliance on the first language in healthcare education, which could impede the development of proficiency in medical English, particularly when dealing with specialized terminologies. Without a carefully balanced approach, translanguaging might unintentionally reinforce language barriers rather than dismantle them.

To maximize the benefits of translanguaging and minimize its risks, institutions must adopt a strategic and evidence-based approach. The findings suggest that a one-size-fits-all approach to translanguaging is unlikely to succeed. Instead, educators should tailor their practices to the specific needs of their students, considering factors such as linguistic backgrounds, levels of English proficiency, and professional aspirations [13, 15]. A well-structured translanguaging strategy encourages the development of bilingual proficiency, enabling students to effectively navigate multilingual healthcare environments. Moreover, institutional support is critical for the successful implementation of translanguaging. Without adequate investment in training, resources, and policy development, translanguaging risks becoming an underutilized strategy that fails to achieve its full potential. Institutions must prioritize the creation of supportive environments that empower educators and students alike to embrace translanguaging as a valuable tool for improving communication and cultural competence.

### Implications

The study has significant implications for healthcare education, particularly in multilingual and multicultural contexts. The evidence from both primary and secondary data underscores the importance of adopting a flexible, inclusive approach to language use in healthcare training programs [3]. Educators and policymakers need to recognize the diverse linguistic backgrounds of their students and integrate translanguaging strategies to leverage students’ linguistic resources. This approach requires a shift away from rigid, monolingual teaching practices and a move toward a more dynamic model of language use that values and incorporates multilingualism [14]. In practice, this means creating learning environments that allow students to comfortably use their full linguistic repertoire to engage with course materials, participate in discussions, and demonstrate their understanding of complex medical concepts. For such an approach to be effective, translanguaging must be thoughtfully incorporated into the curriculum with clear guidelines and robust support for both students and educators [24]. This also involves training healthcare educators to be proficient in translanguaging and equipping them with the necessary skills to facilitate a culturally competent learning experience.

The findings also imply the need for institutional support in adopting translanguaging. Many educators and administrators may resist implementing translanguaging due to concerns about complexity or impracticality, particularly in resource-constrained healthcare settings [15]. To overcome this resistance, institutions must invest in training programs that equip educators with a solid understanding of translanguaging strategies and their practical applications. Additionally, healthcare institutions should allocate resources to develop multilingual educational materials, recruit trained multilingual staff and provide ongoing professional development opportunities. Training programs should develop competencies in bilingual clinical communication, cultural competence, and the ability to navigate multilingual healthcare environments. These programs must strike a balance between leveraging students’ native linguistic resources, such as Arabic, and ensuring proficiency in English, which remains the dominant language in global medical discourse. Curricular modifications could include integrating translanguaging strategies into clinical simulations, offering bilingual instructional materials, and providing targeted support for English medical terminology acquisition. Such adjustments would address identified weaknesses, such as over-reliance on the first language, while capitalizing on strengths like enhanced comprehension and cultural responsiveness.

The broader implications of this study extend beyond healthcare education to other fields where multilingualism and cultural diversity are central concerns. Translanguaging represents a shift toward more inclusive and socially just language practices that challenge traditional, monolingual norms. By embracing translanguaging, educational institutions can not only improve communication and cultural competence but also promote greater equity and inclusivity in educational and professional settings [30].

## Conclusion

This study explored the role of translanguaging in healthcare education. It highlights its potential to enhance communication and foster cultural competence, as well as challenges associated with it, such as over-reliance on the first language and resistance from educators and institutions. Despite threats and limitations, the findings show that it can bridge language gaps, promote inclusivity, and improve patient-provider interactions. The study underscores a transformative educational value of translanguaging, not merely as a linguistic strategy but as a pedagogical tool. In healthcare education, translanguaging fosters deeper engagement, cultural responsiveness, and more authentic clinical communication. It can be adopted to empower multilingual learners to navigate complex clinical interactions with greater confidence and clarity.

### Limitations and further research

Although the findings offer valuable insights, there are some limitations that should be pointed out. A major limitation is due to the small sample size. The non-significant finding at *p* = 0.065 represents a non-significant trend, and further research with larger cohorts may determine whether this pattern reflects a meaningful effect. Also, the sample was drawn from a gender-segregated educational setting, which is standard in Saudi medical education, and reflects the institutional reality within which the study was conducted. It gives way for future research to explore the phenomena in female educational settings. The study is also limited by its cross-sectional design, reliance on self-reported data, and the specific institutional context in which it was conducted. These factors may affect the generalizability of the results across different clinical settings and cultural contexts. To address the limitations and widen the spectrum of investigation, future research may consider longitudinal designs to explore how translanguaging during training influences actual clinical communication over time.

Expanding the research across diverse institutions and healthcare systems would help validate and refine the pedagogical implications of translanguaging in clinical education. Extended research could explore the long-term impact of translanguaging on students’ professional development, particularly within specific healthcare fields such as nursing, dentistry, and public health. To deepen understanding of translanguaging’s influence on communication, care delivery, and professional growth in multilingual healthcare settings, it would be valuable to investigate patients’ perspectives. Such research could reveal how translanguaging affects their experiences, satisfaction, and health outcomes.

## Supplementary Information

Supplementary Material 1.

## Data Availability

Data can be made available at reasonable request to the corresponding author.
