# Supplementary material for: A SWOT analysis of translanguaging in healthcare education: overcoming language barriers for future professionals
Source: BMC Med Educ. 2025 Dec 12;26:104. doi: 10.1186/s12909-025-08271-8 (PMC12822034; doi:10.1186/s12909-025-08271-8)
Supplement: Supplementary file 1 — Supplementary Material 1. [file 12909_2025_8271_MOESM1_ESM.docx]

**Appendix A: Survey Instrument**

**Participants responded to each item using the following 5-point Likert scale:

(1) Strongly Agree (2) Agree (3) Neutral (4) Disagree (5) Strongly Disagree**

Q1: Using Arabic alongside English in healthcare education enhances students' comprehension of complex medical concepts and specific terminology.

( ) 1 ( ) 2 ( ) 3 ( ) 4 ( ) 5

Q2: Using Arabic for medical management and instructions in healthcare education provides an opportunity to support students' learning without language barriers.

( ) 1 ( ) 2 ( ) 3 ( ) 4 ( ) 5

Q3: Relying on Arabic for answering oral questions during healthcare education may limit students' ability to develop medical communication skills in English.

( ) 1 ( ) 2 ( ) 3 ( ) 4 ( ) 5

Q4: Allowing students to use Arabic for written communication could hinder their proficiency in writing medical terms in English.

( ) 1 ( ) 2 ( ) 3 ( ) 4 ( ) 5

Q5: Using Arabic in group discussions during medical classes improves peer collaboration and teamwork.

( ) 1 ( ) 2 ( ) 3 ( ) 4 ( ) 5

Q6: The use of Arabic in healthcare education supports students’ confidence when speaking in clinical scenarios.

( ) 1 ( ) 2 ( ) 3 ( ) 4 ( ) 5

Q7: Incorporating Arabic in using medical terms enhances students' motivation to learn and engage more deeply with the material.

( ) 1 ( ) 2 ( ) 3 ( ) 4 ( ) 5

Q8: Integrating Arabic in medical training improves students' overall understanding of the subject matter and course content.

( ) 1 ( ) 2 ( ) 3 ( ) 4 ( ) 5

Q9: Excessive use of Arabic in medical interns may limit students’ ability to master medical terminologies in English, potentially affecting their professional communication skills.

( ) 1 ( ) 2 ( ) 3 ( ) 4 ( ) 5

Q10: Students’ comfort in using Arabic may reduce their willingness to engage with English, which could be a disadvantage in their future medical careers where English is dominant.

( ) 1 ( ) 2 ( ) 3 ( ) 4 ( ) 5
